# Resveratrol promotes MICA/B expression and natural killer cell lysis of breast cancer cells by suppressing c-Myc/miR-17 pathway

**DOI:** 10.18632/oncotarget.19445

**Published:** 2017-07-22

**Authors:** Jie Pan, Jiaying Shen, Wengong Si, Chengyong Du, Danni Chen, Liang Xu, Minya Yao, Peifen Fu, Weimin Fan

**Affiliations:** ^1^ Program of Innovative Cancer Therapeutics, Division of Hepatobiliary and Pancreatic Surgery, Department of Surgery, The First Affiliated Hospital, College of Medicine, Zhejiang University, Zhejiang Province, Hangzhou 310003, China; ^2^ Breast Center, The First Affiliated Hospital, College of Medicine, Zhejiang University, Zhejiang Province, Hangzhou 310003, China; ^3^ Clinical Research Center, The First Affiliated Hospital, College of Medicine, Zhejiang University, Zhejiang Province, Hangzhou 310003, China; ^4^ Department of Pathology and Laboratory Medicine, Medical University of South Carolina, Charleston, SC 29425, USA

**Keywords:** resveratrol, NKG2D, miR-17, MICA, c-Myc

## Abstract

Major histocompatibility complex class I chain-related proteins A and B (MICA and MICB) are important ligands for recognition of tumor cells by immune effector cells. Here, we report that resveratrol upregulated the protein and mRNA expression of MICA and MICB in breast cancer cells, which in turn promoted breast cancer cell lysis by natural killer (NK) cells *in vitro* and *in vivo*. Antibodies against NK group 2 member D blocked this effect. The 3′-untranslated regions of *MICA* and *MICB* were found to be direct binding targets of miR-17. MICA and MICB expression increased or decreased in breast cancer cells transfected with a miR-17 inhibitor or mimic, respectively. *C-Myc* overexpression/knockdown increased/decreased transcription of the *miR-17-92* cluster host gene. Resveratrol suppressed c-Myc expression, which inhibited the transcription of *miR-17-92* cluster, thereby downregulating miR-17. MiR-17 expression correlated inversely with *MICA* and *MICB* expression and overall survival in two sets of breast cancer specimens. Resveratrol thus upregulates MICA and MICB by suppressing the c-Myc/miR-17 pathway in breast cancer cells, and increases the cytolysis of breast cancer cells by NK cells. This suggests resveratrol has the potential to promote antitumor immune responses in breast cancer patients.

## INTRODUCTION

Immune escape is one of the main reasons for the rapid progression of cancer and the poor efficacy of immunotherapy. The inactivation of natural killer (NK) cells contributes to the immune escape of tumor cells. NK cells serve as an antitumor defense through their direct cytotoxic and indirect immune-regulatory capacities. The NK cell activation is controlled by signals derived from the ligation of activating receptors. Natural killer group 2 member D (NKG2D) is one of the most prominent activating receptors of NK cells [1]. In humans, the NKG2D ligands (NKG2DLs) comprise eight surface glycoproteins, including the major histocompatibility complex class I chain-related proteins A and B (MICA/B) and the UL16-binding protein family. These NKG2DLs are considered to be promising targets by which the immunogenicity of cancer cells can be improved [2]. While NKG2DL expression is low or undetectable on normal cells, it can be induced during stress and malignant transformation. However, some malignantly transformed cells can still evade immune detection and elimination by reducing their surface levels of NKG2DLs by various mechanisms, such as by downregulating the expression of these proteins [3] and/or shedding them from the cell surface [4].

To this end, many researchers have aimed to induce the expression of NKG2DLs with agents such as decitabine, bortezomib, suberoylanilide hydroxamic acid, doxorubicin and melphalan [5-8]. However, most of these agents are antitumor drugs with inevitable side effects, so it would be meaningful to identify safer agents. The regulation of NKG2DLs by dietary compounds has been investigated to a lesser extent. Resveratrol, a naturally occurring dietary compound, has been suggested to enhance the immune response to certain cancers, including leukemia, lung cancer and hepatic cancer [9-11]. Of particular interest, resveratrol was reported to upregulate NKG2DLs by activating the ataxia–telangiectasia mutated kinase (ATM) in leukemia cells [10]. However, the mechanism whereby resveratrol upregulates NKG2DLs is not fully understood. On the other hand, it is known that the levels of some NKG2DLs are regulated by cancer-relevant microRNAs (miRNAs), such as miR-20a, miR-34a, miR-34c, miR-106b, miR-93, miR-373, and miR-520 [12-15].

In the current study, we hypothesized that resveratrol could upregulate MICA/B expression (and, in turn, cancer cell lysis by NK cells) by altering miRNA expression, and provided experimental validation for this hypothesis in human breast cancer. In examining the mechanism whereby resveratrol regulates MICA/B, we discovered a novel c-Myc/miR-17 pathway that governs MICA/B expression in breast cancer.

## RESULTS

### Resveratrol increases MICA and MICB expression in breast cancer cell lines and xenograft tumors

**Figure 1:**
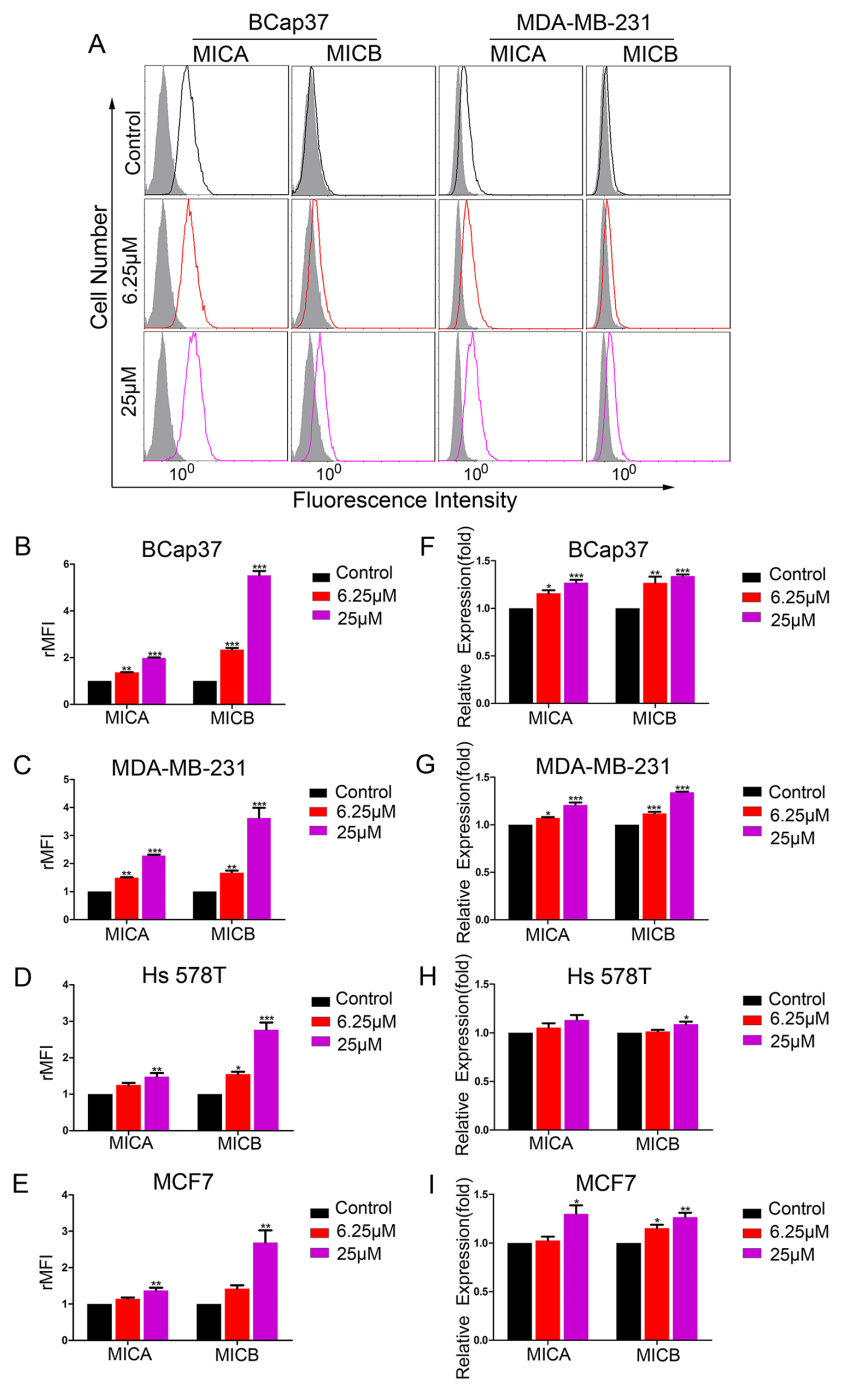
Resveratrol upregulates MICA and MICB expression in breast cancer cells *in vitro* Breast cancer cell lines were treated with resveratrol (6.25 μM or 25 μM) or the control for 48 h. **(A-E)** The protein levels of MICA and MICB on BCap37, MDA-MB-231, Hs 578T and MCF-7 cells were detected by flow cytometry. (A) depicts representative results from (B) and (C). **(F-I)** The mRNA levels of *MICA* and *MICB* were detected by qRT-PCR, with *HPRT1* as a reference. Error bars represent the SEM obtained from three independent experiments. *P<0.05, **P<0.01, ***P<0.001.

To determine whether resveratrol could increase the expression of MICA and MICB in breast cancer cells, we exposed BCap37, MDA-MB-231, Hs 578T and MCF-7 cells to 6.25 μM or 25 μM resveratrol for 48 h. The surface-protein and mRNA levels of MICA and MICB were detected by flow cytometry and qRT-PCR, respectively. As shown in Figure 1, resveratrol treatment increased the surface levels of MICA and MICB on breast cancer cells in a dose-dependent manner (Figure 1A-1E). In parallel, the mRNA levels of *MICA* and *MICB* were also upregulated (Figure 1F-1I). The upregulation was most obvious in the BCap37 and MDA-MB-231 cell lines. A 48-h cell viability assay revealed that, at a resveratrol concentration of 25 μM, the cell viability was 98.3±1.9% in MDA-MB-231 cells and 92.9±2.9% in BCap37 cells (Supplementary Figure 1A). In an apoptosis assay, 25 μM resveratrol induced apoptosis in less than 4% of cells (Supplementary Figure 1B). Thus, at the concentrations adopted in this study, the direct toxicity of resveratrol to the target cells was limited.

**Figure 2:**
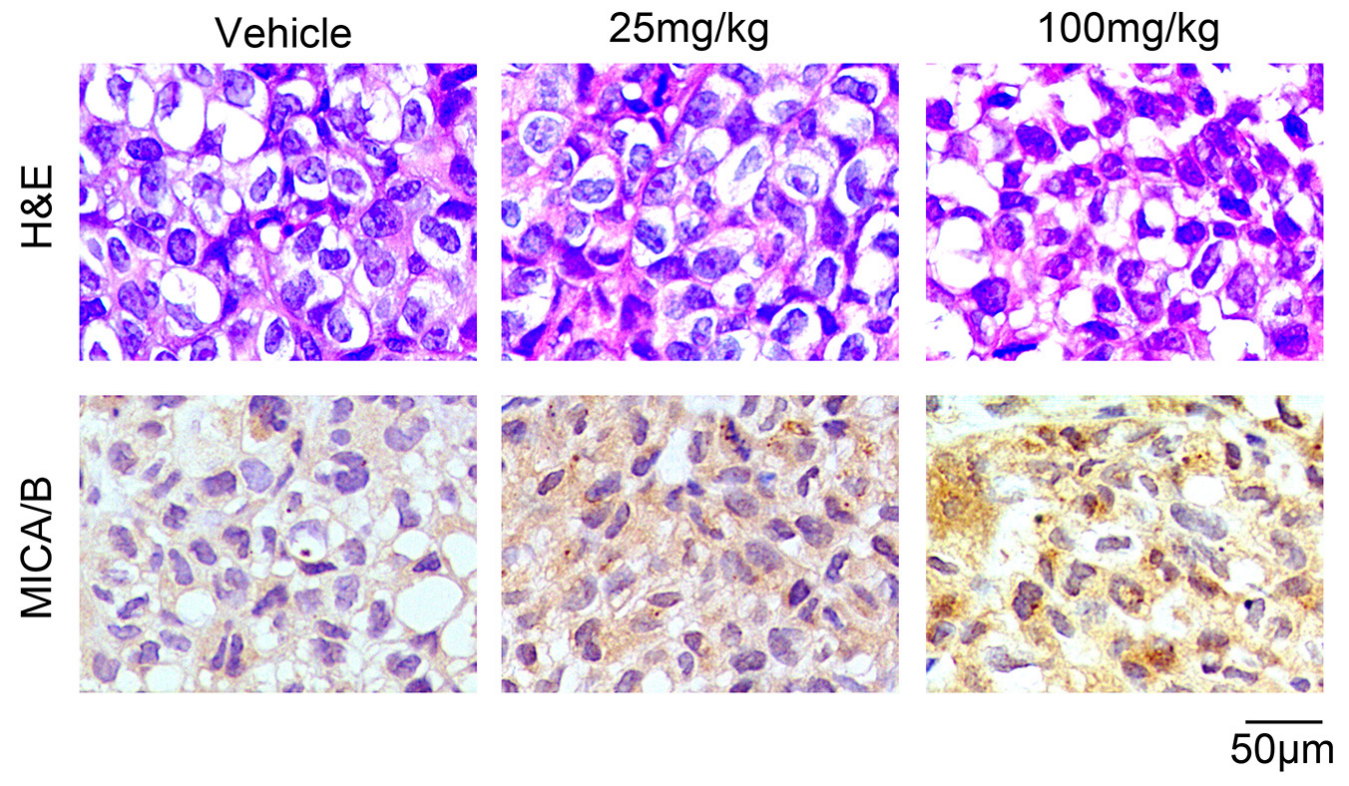
Resveratrol upregulates MICA/B expression in breast cancer xenograft tumors Representative images from hematoxylin & eosin staining and an immunohistochemistry assay. BCap37 cells were subcutaneously implanted into the right hind flanks of female BALB/c (nu/nu) mice. Tumor tissues were collected after 28-day administration of resveratrol or vehicle. Paraffin sections of the tumor samples were prepared, and the cellular morphology was detected by hematoxylin & eosin staining (upper). MICA/B expression was examined with an immunohistochemistry assay (lower) (magnification ×400).

We further explored the effects of resveratrol *in vivo*. BCap37 cells were subcutaneously implanted into the right hind flanks of female BALB/c (nu/nu) mice to form xenograft tumors, and resveratrol was injected intraperitoneally at different doses (0, 25 or 100 mg/kg/d) for 28 days. In line with the results *in vitro*, resveratrol dose-dependently increased the expression of MICA/B in xenograft tumors (Figure 2). Moreover, at the endpoint of the study, all the mice were alive and in good condition, and their body weights did not differ significantly (Supplementary Figure 2A), indicating that 100-mg/kg/d resveratrol treatment for 28 days was safe for mice. We also found that resveratrol directly reduced tumor growth, as has been reported by other researchers (Supplementary Figure 2B, 2C) [16, 17]. These data indicate that resveratrol can upregulate MICA and MICB in breast cancer cells *in vitro* and *in vivo*.

### Resveratrol increases the lysis of breast cancer cells by NK cells by promoting NKG2D/NKG2DL binding *in vitro*

**Figure 3:**
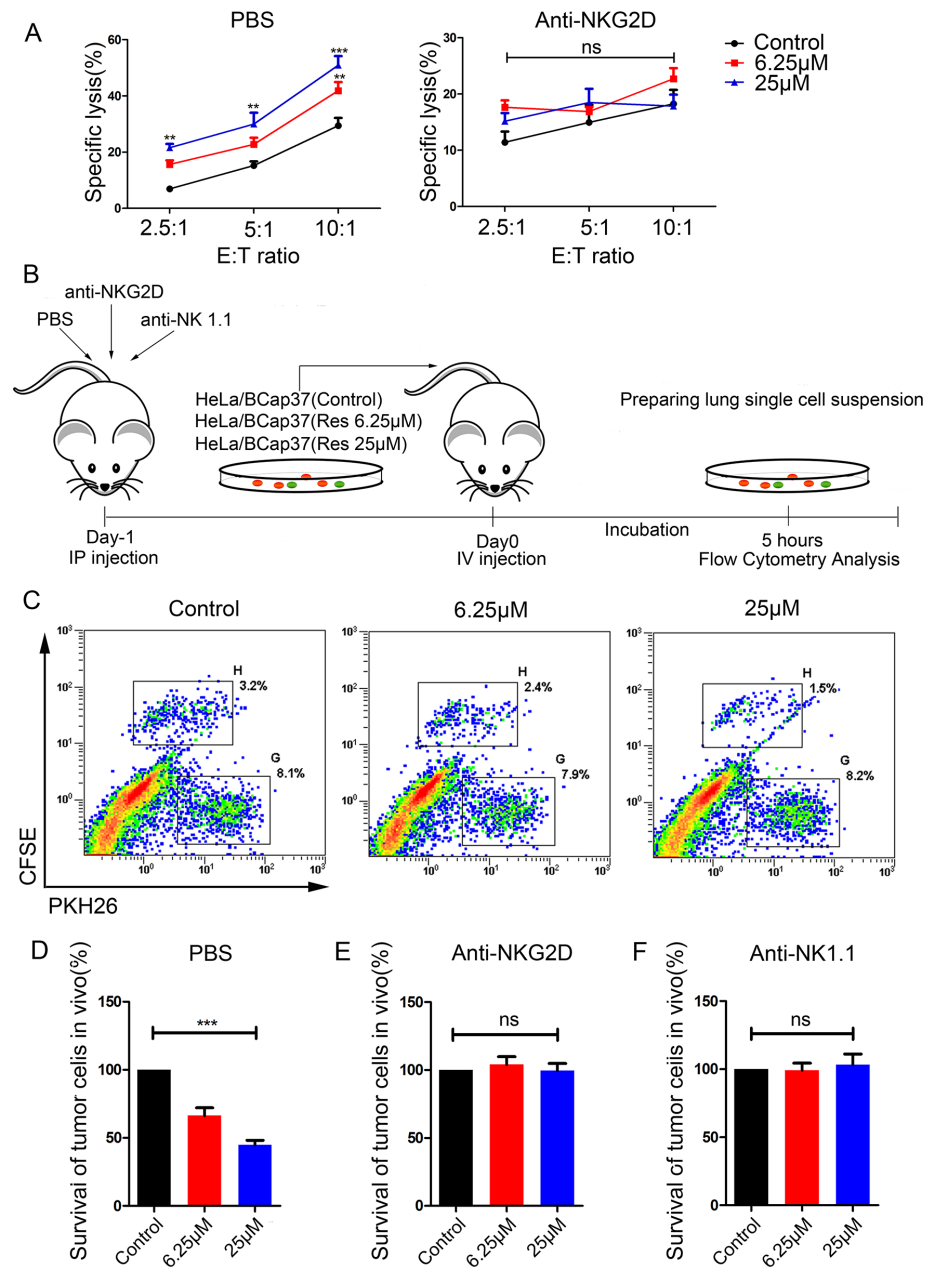
Resveratrol increases the susceptibility of breast cancer cells to cytotoxicity *in vitro* and *in vivo* **(A)** BCap37 cells were exposed to different concentrations of resveratrol for 48 h. NK-92MI cells were pretreated with anti-NKG2D Abs (right) or PBS (left) for 1 h before the cytotoxicity assay. Cytotoxicity assays were performed with NK-92MI cells as effector cells at different effector-to-target ratios. **(B)** Schematic representation of the *in vivo* experimental procedures. **(C-F)** Mice were injected with PBS (C, D), anti-NKG2D (E) or anti-NK 1.1 (F) 24 h before being injected with a mixture of BCap37 and HeLa cells. Flow cytometry was used to analyze the ratios of BCap37 cells to HeLa cells in lung single-cell suspensions. (C) depicts representative results from (D). Error bars represent the SEM obtained from three independent experiments. **P<0.01, ***P<0.001. Abbreviations: IP, intraperitoneal; IV, intravenous.

We next examined whether resveratrol could increase the susceptibility of breast cancer cells to lysis by NK cells. Bcap37 cells were treated with 6.25 μM or 25 μM resveratrol for 48 h and then used in NK cytotoxicity assays. As shown in Figure 3A, the upregulation of MICA and MICB by resveratrol was functional, as it moderately induced the killing of target cells tested at various effector-to-target ratios (Figure 3A, left). Furthermore, resveratrol-enhanced cytotoxicity was caused by increasing NKG2D recognition, as pretreatment of NK cells with an anti-NKG2D monoclonal antibody (mAb) resulted in equivalent killing of all targets (Figure 3A, right). Thus, resveratrol upregulates MICA and MICB and promotes NKG2D recognition to induce the death of target cells by NK cells.

### Resveratrol contributes to immune clearance *in vivo*

To further explore the potential effects of resveratrol on breast cancer cell clearance *in vivo*, we performed experiments with C57BL/6 mice. The murine NKG2D receptor recognizes the human ligands and *vice versa*, though the human and mouse NKG2D ligands differ [18, 19]. A short-term *in vivo* assay (illustrated in Figure 3B) was adopted, and HeLa cells were used as an internal control.

In accordance with the results of our *in vitro* experiment, the survival rate of resveratrol-treated BCap37 cells injected into mice declined dose-dependently (Figure 3C, 3D). That is, as higher concentrations of resveratrol were used to treat the BCap37 cells, more BCap37 cells were cleared by the murine immune system and fewer carboxyfluorescein succinimidyl ester (CFSE)-labeled BCap37 cells were detected by flow cytometry. To explore whether the enhanced immune clearance was due to the binding between NKG2D and NKG2DLs, we intraperitoneally injected mice with anti-NKG2D or anti-NK1.1 mAbs to block the related receptors. The *in vivo* blocking of NKG2D activity with the anti-NKG2D mAb (Figure 3E) abolished the effects of resveratrol that had been observed in the phosphate-buffered saline (PBS) control group (Figure 3C, 3D). NK cells were the main effectors of the clearance, as the depletion of NK cells with the anti-NK1.1 mAb completely abolished the effects of resveratrol (Figure 3F). Thus, the upregulation of MICA and MICB by resveratrol promotes the immune recognition and clearance of tumor cells *in vivo*.

### Resveratrol reduces the expression of miR-17

**Figure 4:**
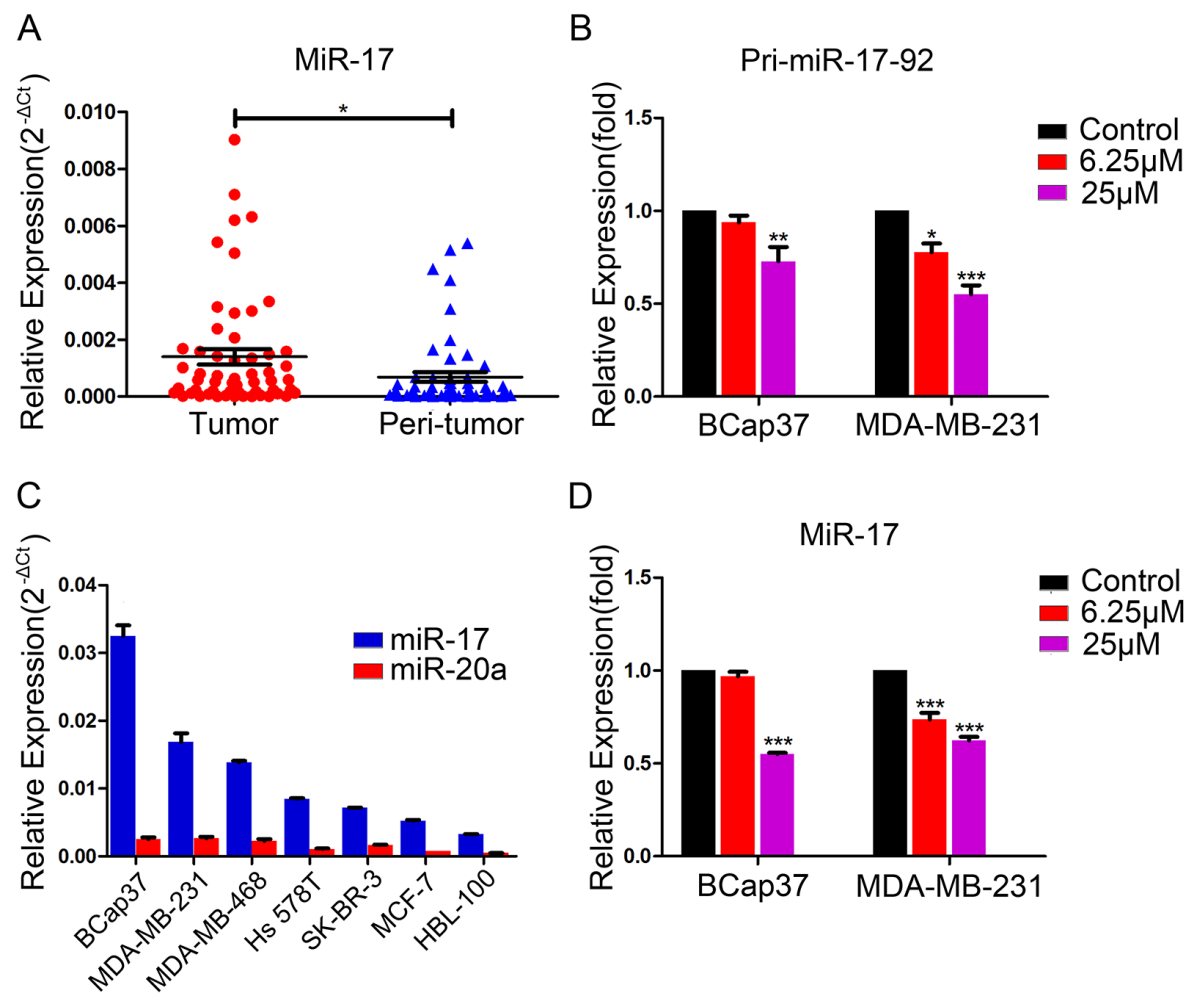
Endogenous expression of miR-17 in breast cancer cells and suppression of *pri-miR-17-92* and miR-17 by resveratrol **(A)** Endogenous miR-17 levels in breast tumor tissues and non-tumorous tissues from 56 patients were detected by qRT-PCR, with *U6* as a reference. **(B)** BCap37 and MDA-MB-231 cells were pretreated with vehicle or different concentrations of resveratrol for 48 h. Transcription of the *miR-17-92* cluster was detected by qRT-PCR, with *HPRT1* as a reference. **(C)** The endogenous levels of miR-17 and miR-20a in six breast cancer cell lines and one normal breast cell line were assessed by qRT-PCR, with *U6* as a reference. **(D)** BCap37 and MDA-MB-231 cells were treated as in (B). MiR-17 levels were assessed by qRT-PCR, with *U6* as a reference. Data represent the mean ± SEM of three independent experiments. *P<0.05, **P<0.01, ***P<0.001.

The *miR-17-92* cluster, which is processed from the transcript of Chromosome 13 open reading frame 25 (C13orf25), consists of six microRNAs: miR-17, miR-18a, miR-19a, miR-20a, miR-19b, and miR-92a [7]. This cluster, known as oncomiR-1, is always overexpressed in tumors. We analyzed 56 pairs of breast tumor and peri-tumor specimens, and found that miR-17 expression was higher in breast tumor tissues than in non-tumorous tissues (Figure 4A). To determine whether resveratrol could suppress the expression of the *miR-17-92* cluster, we examined the expression of this gene cluster in BCap37 and MDA-MB-231 cells pretreated with resveratrol. When breast cancer cells were treated with resveratrol (6.25 μM or 25 μM) for 48 h, the transcription of the *miR-17-92* cluster declined dose-dependently (Figure 4B).

Next, using various databases, we tabulated the predicted binding scores of *miR-17-92* cluster members to the *MICA/B* 3′-UTRs, and calculated the aggregate binding scores from these databases (Supplementary Tables 1, 2). MiR-17 and miR-20a received the highest scores, so we chose these two miRNAs for further experiments. We evaluated the expression of miR-17 and miR-20a in six different breast cancer cell lines (BCap37, MDA-MB-231, MDA-MB-468, Hs 578T, SK-BR-3, and MCF-7) and one normal breast cell line (HBL-100) by qRT-PCR. As shown in Figure 4C, these breast cancer and normal cells expressed miR-17 and miR-20a at different levels. The expression of miR-17 was higher than that of miR-20a in both breast cancer and normal cells, and was higher in BCap37 and MDA-MB-231 cells than in the other cell lines. Considering this, along with the fact that miR-17 and miR-20a belong to the same microRNA family and share the same seed sequence, we focused on miR-17 expression in BCap37 and MDA-MB-231 cells in the following experiments. When the expression of miR-17 was investigated in BCap37 and MDA-MB-231 cells treated with resveratrol at various concentrations for 48 h, resveratrol was found to downregulate miR-17 dose-dependently (Figure 4D). These data indicate that resveratrol suppresses the transcription of the *miR-17-92* cluster and subsequently downregulates miR-17.

### MiR-17 downregulates *MICA* and *MICB* expression by binding to their 3′-untranslated regions (3′-UTRs)

**Figure 5:**
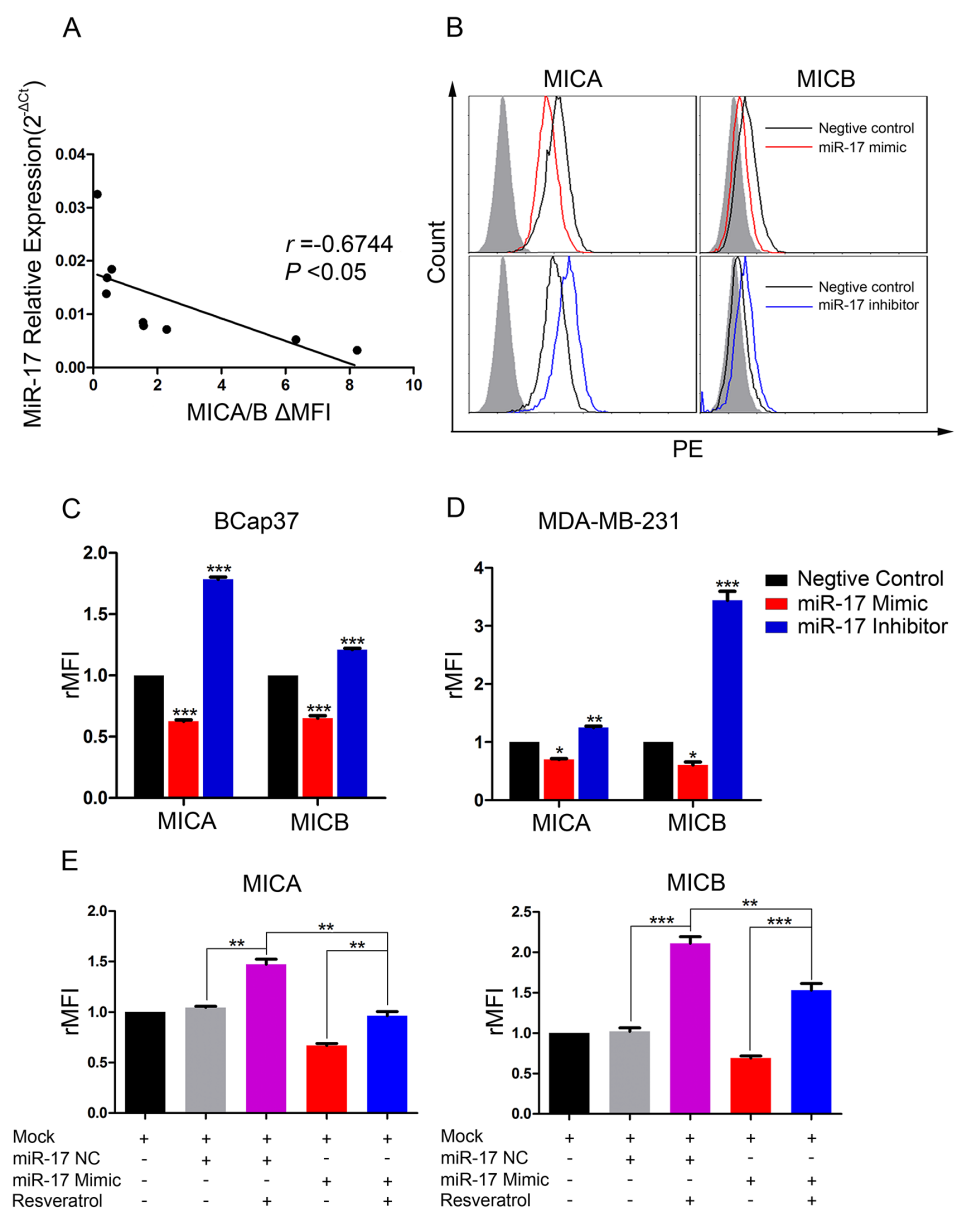
MiR-17 downregulates MICA and MICB expression, which can be blocked by resveratrol **(A)** MiR-17 and MICA/B levels in seven breast cancer cell lines and two normal breast cell lines were assessed by qRT-PCR and flow cytometry, respectively. The correlation between the two was analyzed by linear regression. **(B-D)** BCap37 (B, C) and MDA-MB-231 (D) cells were transfected with a 50 nM miR-17 mimic, inhibitor, or the corresponding NC for 24 h. The surface levels of MICA and MICB were detected by flow cytometry 72 h after transfection. (B) depicts representative results from (C). **(E)** BCap37 cells transfected with a miR-17 mimic or NC for 24 h were treated with culture medium or 25 μM resveratrol for another 48 h. Then, the levels of MICA and MICB on cells were detected by flow cytometry. Error bars represent the SEM obtained from three independent experiments. *P<0.05, **P<0.01, ***P<0.001.

We next assessed the endogenous expression of miR-17 and MICA/B in seven breast cancer cell lines (BCap37, MDA-MB-231, MDA-MB-468, Hs 578T, SK-BR-3, BT-474 and MCF-7) and two normal breast cell lines (HBL-100 and Hs 578Bst). MiR-17 levels correlated negatively with MICA/B levels in all the tested cell lines (Figure 5A). To further explore the regulation of MICA and MICB expression by miR-17, we transiently transfected BCap37 and MDA-MB-231 cells with a miR-17 mimic, miR-17 inhibitor, or the corresponding negative control (NC). MICA and MICB were upregulated in miR-17 inhibitor-transfected BCap37 cells, and were downregulated in miR-17 mimic-transfected BCap37 cells (Figure 5B, 5C). Similar observations were made in MDA-MB-231 cells (Figure 5D).

To evaluate whether resveratrol could prevent the downregulation of MICA and MICB by miR-17, we transiently transfected BCap37 cells with the miR-17 mimic or NC for 24 h and then treated them with 25 μM resveratrol for another 48 h. As shown in Figure 5E, the miR-17 mimic downregulated MICA and MICB, but resveratrol exposure remarkably blocked this downregulation.

**Figure 6:**
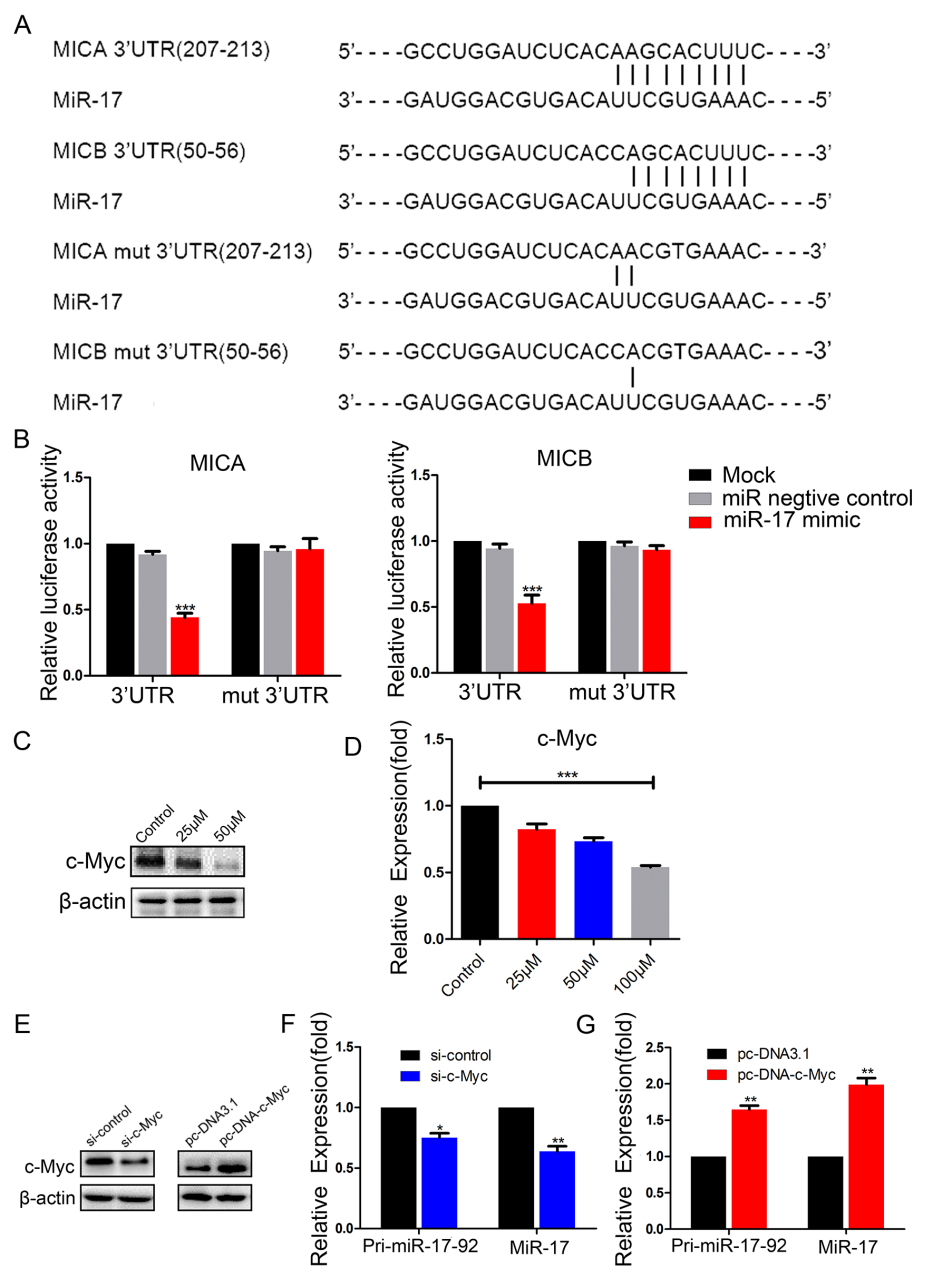
MiR-17 binds to the 3′-UTRs of *MICA* and *MICB*, and c-Myc promotes the expression of miR-17 **(A)** Schematic representation of predicted miR-17 binding sites in the 3′-UTRs of *MICA/B,* and 3′-UTR mutated alignment. **(B)** Luciferase reporter assay of BCap37 cells co-transfected with a miR-17 mimic, NC or mock and either wild-type or mutated luciferase plasmid. **(C, D)** BCap37 cells were treated with different concentrations of resveratrol for 48 h. The expression of c-Myc was detected by Western blotting (C) and qRT-PCR (D). **(E)** c-Myc protein levels in BCap37 cells transfected with control siRNA (si-control) or *c-Myc* siRNA (si-c-Myc) (left) and with pc-DNA3.1 or pc-DNA-c-Myc (right) were analyzed by Western blotting. β-actin is shown as a loading control. **(F, G)** *Pri-miR-17-92* and miR-17 levels were evaluated in *c-Myc*-knockdown (F) or *c-Myc*-overexpressing (G) BCap37 cells by qRT-PCR. Error bars represent the SEM obtained from three independent experiments. *P<0.05, **P<0.01, ***P<0.001.

To determine whether miR-17 directly targeted *MICA* and *MICB*, we performed luciferase reporter assays in BCap37 cells. Wild-type 3′-UTRs of *MICA* and *MICB* and mutated sequences (mut-MICA and mut-MICB) were individually cloned into reporter plasmids (psiCHECK-2) downstream of the Renilla luciferase gene (Figure 6A). BCap37 cells were transfected with a reporter plasmid only, or co-transfected with the reporter plasmid and either 50 nM miR-17 mimic or 50 nM NC. The luciferase activities of the reporters with wild-type *MICA* or *MICB* 3′-UTRs were significantly suppressed by miR-17 mimic transfection, while the activities of the reporters with mutant *MICA* or *MICB* 3′-UTRs did not change significantly (Figure 6B). These results demonstrate that miR-17 binds directly to *MICA* and *MICB*.

Taken together, these findings suggest that miR-17 downregulates *MICA* and *MICB* by directly binding to their 3′-UTRs, while resveratrol can block this downregulation.

### Resveratrol downregulates miR-17 expression by suppressing c-Myc

To determine how resveratrol downregulated miR-17, we focused on the transcription factor c-Myc, which has been reported to bind directly to the *miR-17-92* cluster genomic locus in human lymphoma cells [20]. We previously found that there were c-Myc binding sites in the *miR-17-92* cluster transcriptional initiation site in breast cancer cells, and that c-Myc promoted the transcription of this gene cluster (Wengong Si et al., revised).

Here, we hypothesized that c-Myc was involved in the regulation of *miR-17-92* cluster expression by resveratrol. To test this hypothesis, we investigated the effect of resveratrol on c-Myc expression in BCap37 cells. Following 48-h resveratrol treatment, c-Myc mRNA and protein levels decreased dose-dependently (Figure 6C, 6D).

To determine the effect of c-Myc on the transcription of the *miR-17-92* cluster, we transfected BCap37 cells with *c-Myc* siRNA or overexpression plasmids to knock down or overexpress *c-Myc* (Figure 6E). Knockdown of *c-Myc* reduced the expression of *pri-miR-17-92* and miR-17 (Figure 6F). On the other hand, overexpression of *c-Myc* significantly increased *pri-miR-17-92* and miR-17 expression (Figure 6G). These observations suggest that resveratrol reduces miR-17 expression by suppressing c-Myc.

### MiR-17 expression correlates inversely with *MICA/B* expression and overall survival in breast cancer patients

**Figure 7:**
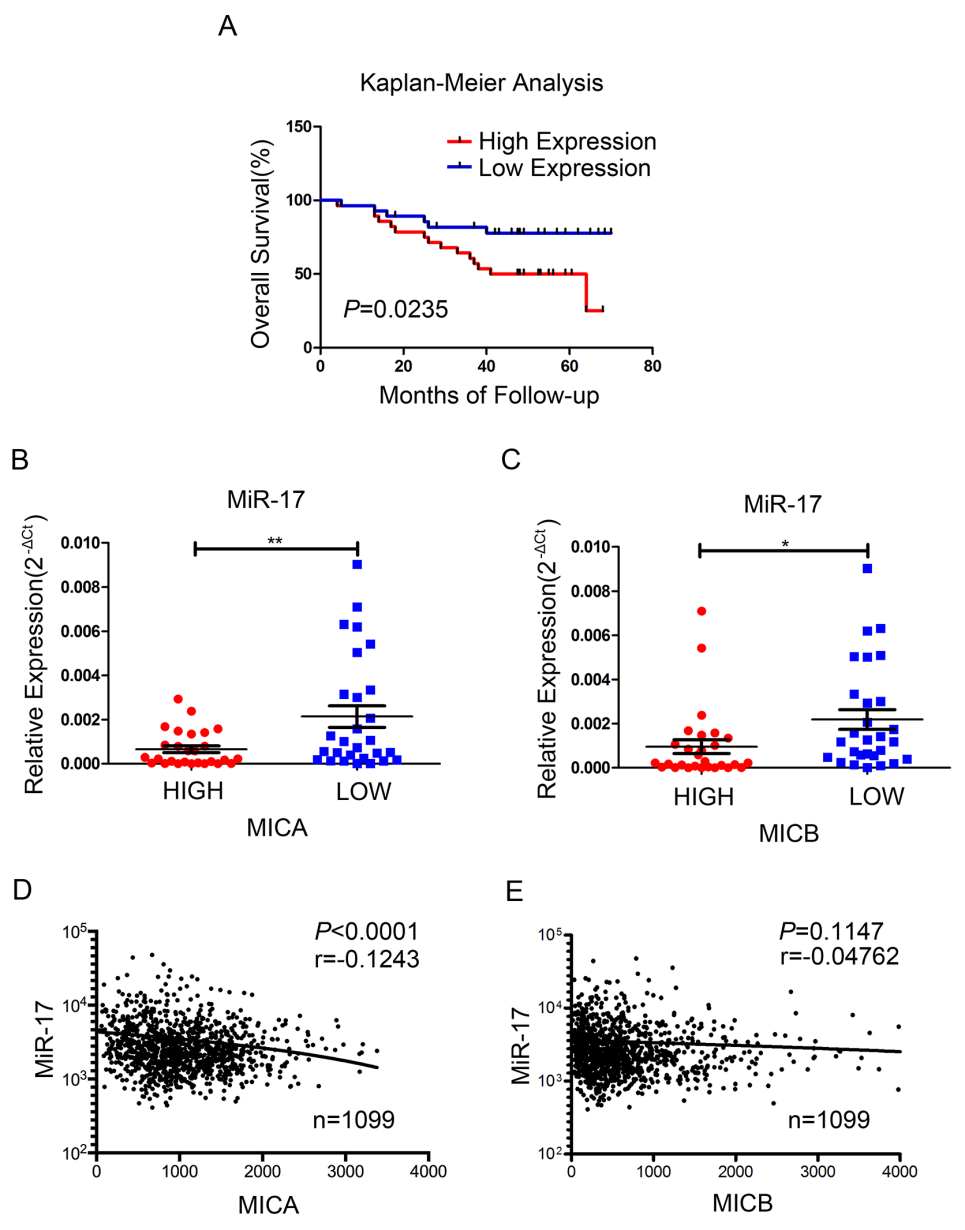
Association of miR-17 with the expression of *MICA/B* and survival in human breast cancer **(A-C)** Data from 56 clinical breast cancer patients. (A) Kaplan-Meier overall survival curves comparing high and low miR-17 expression groups; P value was based on a log-rank test. (B, C) The expression of miR-17 was compared in high and low *MICA* (B) or *MICB* (C) expression groups. **(D, E)** The association of miR-17 expression with *MICA* (D) and *MICB* (E) expression in 1099 breast cancer samples from TCGA. Error bars in B and C represent the mean ± SEM. *P<0.05, **P<0.01.

To investigate the significance of miR-17 in breast cancer patients, we analyzed the correlations of miR-17 expression with *MICA/B* expression, disease stage, overall survival and cancer recurrence. The levels of miR-17 and *MICA/B* were evaluated in 56 clinical breast cancer specimens. The median relative level of miR-17 or *MICA/B* was adopted as the threshold value, such that miRNA or mRNA levels above or below this level were considered as high or low expression, respectively. In line with other studies [21], the overall survival probability was poorer and the cancer recurrence rate was higher in patients with higher levels of miR-17 than in those with lower levels (Figure 7A, Supplementary Table 3). The expression of miR-17 correlated inversely with the expression of *MICA* and *MICB* in the 56 specimens (Figure 7B, 7C). These specimens were then divided into three groups according to the WHO staging system (stage I, II, and III). A trend for higher miR-17 expression was observed in late-stage patients (Supplementary Figure 3).

Because of our small sample size, we further analyzed 1099 available breast cancer samples from The Cancer Genome Atlas (TCGA). In these samples, miR-17 expression correlated negatively with *MICA* expression, but did not correlate significantly with *MICB* expression (Figure 7D, 7E).

Thus, higher miR-17 levels correlate with a poorer overall survival probability, higher cancer recurrence rate and lower *MICA/B* level in breast cancer patients.

## DISCUSSION

NKG2D-NKG2DL binding is important in the antitumor immune response, as it ensures innate immunity to tumor cells and co-stimulates T-cell subset activation [22, 23]. High levels of NKG2DLs are associated with the increased cytotoxic activity of effector cells [24]. However, tumor cells have developed various strategies to downregulate NKG2DLs, allowing them to avoid immune recognition and clearance. Thus, the NKG2DL system has attracted vivid interest as a potential target of treatments to improve antitumor immune responses [2].

Functional analyses in this study demonstrated that resveratrol increased the susceptibility of breast cancer cells to lysis by NK cells *in vitro* and *in vivo* by inducing MICA and MICB expression. Resveratrol is a natural polyphenolic flavonoid compound with multiple health-promoting effects, mainly against oxidative stress and inflammation. Resveratrol is produced by many commonly consumed plants, including grapes, apples, raspberries, blueberries, plums and peanuts [11]. Consecutive high-dose administration of resveratrol (up to 5.0 g daily for 29 days) was verified to be safe and well-tolerated in healthy volunteers [25, 26]. In our animal study, we found that consecutive administration of 100 mg/kg/d resveratrol for 28 days was safe in mice. Thus, resveratrol may be a safer candidate than other NKG2DL-inducing agents such as proteasome inhibitors, histone deacetylases and some chemotherapeutic drugs [27-29].

The regulation of MICA and MICB expression is a complex process that involves various transcriptional and post-transcriptional mechanisms. Numerous signals and pathways have been identified as regulators of NKG2DLs, including the ATM (ataxia-telangiectasia, mutated) and ATR (ATM and Rad3-related) pathways, the NF-κB pathway, heat shock factor-1, Sp-family transcription factors and the ERK signaling pathway [4, 6, 30-32]. Here, we have described a novel c-Myc/miR-17 pathway whereby resveratrol upregulates MICA and MICB.

MiR-17, which belongs to the oncogenic *miR-17-92* cluster, is upregulated in a variety of cancers [33, 34]. We found that miR-17 expression was higher in breast tumor tissues than in peri-tumor tissues, consistent with previous reports. Thus far, miR-17 has been reported to promote tumorigenesis and progression by sustaining cell survival, proliferation, self-renewal, migration and angiogenesis. MiR-17 was found to stimulate cancer genesis and progression by suppressing *P130* and *PTEN* [35, 36]. In addition, miR-17 was reported to impair cancer cell apoptosis by downregulating the pro-apoptotic gene *Bim,* and to impede cell cycle arrest by downregulating the cell cycle inhibitor *p21* [33, 37-40]. Overexpression of miR-17 also improved the invasion and migration abilities of cancer cells [41-43]. MiR-17 may promote cancer progression by inducing angiogenesis in solid tumors [44]. Although miR-17 is known to drive tumorigenesis and progression by inhibiting certain tumor suppressors, there has been little evidence of its tumorigenicity from the immunological standpoint. MiR-20a, another component of the *miR-17-92* cluster, was reported to bind to the mRNA of *MICA* and *MICB* [7, 45, 46]. Therefore, we postulated that miR-17 could promote tumor progression by inhibiting MICA/B expression, thus allowing tumor cells to escape from immune recognition.

We discovered that resveratrol suppressed the transcription of the *miR-17-92* cluster host gene and subsequently reduced the expression of miR-17. MICA and MICB expression increased in response to resveratrol treatment, an effect that correlated inversely with miR-17 expression. By performing the dual luciferase reporter assays, miR-17 was verified to reduce *MICA* and *MICB* expression by binding to their 3′-UTRs. These data indicated that resveratrol increased the susceptibility of breast cancer cells to lysis by NK cells through a miR17/MICA/MICB pathway.

However, the mechanism whereby resveratrol suppressed the expression of miR-17 was still unknown. Certain transcription factors, such as c-Myc, p-STAT3 and E2F, are known to transcriptionally activate the *miR-17-92* cluster [7, 20, 47]. C-Myc, which is essential for cell growth, proliferation and tumorigenesis, is dysregulated in a wide range of malignancies, including breast cancer [48, 49]. Our study demonstrated that c-Myc was repressed in resveratrol-treated breast cancer cells. Knockdown or overexpression of *c-Myc* respectively reduced or induced the expression of *miR-17-92*, supporting previous findings that c-Myc could directly bind to the *miR-17-92* cluster promoter [20]. Therefore, we deduced that resveratrol suppressed the expression of c-Myc and consequently inhibited the transcription of *miR-17-92*.

Our findings in breast cancer cells and animal models prompted us to investigate the clinical association of miR-17 with *MICA/B* and its prognostic impact on breast cancer patients. Overexpression of miR-17 has been associated with poor prognosis in certain malignancies [21, 36]. Accordingly, we found that the overall survival was shorter and the cancer recurrence rate was higher in breast cancer patients with higher levels of miR-17. In our analysis of 56 clinical breast cancer specimens and data from TCGA, it was obvious that miR-17 expression correlated inversely with *MICA* and *MICB* expression, potentially indicating that miR-17 inhibited *MICA/B* expression. Though the difference was not significant, patients with late-stage disease were more likely to express higher levels of miR-17 than those with early-stage disease. Thus, high expression of miR-17, which showed a negative correlation with *MICA* and *MICB* levels, might predict a poor prognosis in breast cancer patients.

In conclusion, resveratrol elevated MICA and MICB expression in breast cancer cells by suppressing the c-Myc/miR-17 pathway, thus increasing the susceptibility of breast cancer cells to lysis by NK cells. Therefore, resveratrol treatment could be a feasible way to improve the MICA and MICB mediated anticancer effects in breast cancer patients.

## MATERIALS AND METHODS

### Clinical samples

Paraffin-embedded sections of breast cancer tissues and adjacent non-tumorous breast tissues were derived from 56 patients who underwent surgical resection at the First Affiliated Hospital of Zhejiang University College of Medicine from 2010 to 2011. The respective adjacent non-tumorous breast tissue was used as a reference sample for each cancer tissue. Total RNA (including miRNA) was isolated with the RNeasy® FFPE Kit (Invitrogen, Carlsbad, CA, USA) according to the manufacturer’s protocol, and was stored at −80°C for further study. Clinical parameters, including age, sex, pathological features and TMN stage, were obtained from patients’ medical records. Detailed sample information is listed in Supplementary Table 3. The study protocol was reviewed and approved by the Ethics Committee of Zhejiang University College of Medicine. Written informed consent was obtained from each patient.

### Mice and cell lines

Male C57BL/6 mice (8-9 weeks old) were purchased from Vital River (Beijing, China). Female BALB/c (nu/nu) mice (6 weeks old) were purchased from the Shanghai Laboratory Animal Center (Shanghai, China). All animal experiments were approved by the Ethics Committee of Zhejiang University College of Medicine.

All cell lines were purchased from the Cell Bank of the Chinese Academy of Sciences (Shanghai, China) and maintained according to the manufacturer’s recommendations. Experiments were initiated when the cells exhibited logarithmic growth.

### Cell viability assay

Cell viability was determined by the microculture tetrazolium (MTT) assay. Cells were seeded into 96-well culture plates, incubated for one night, and treated with different concentrations of resveratrol or control medium for 48 h. Four hours prior to the endpoint, the MTT solution was added. The plates were incubated again for 4 h at 37°C in the dark. Then, the medium containing MTT in each well was replaced with 150 μL of dimethyl sulfoxide (DMSO, Sinopharm Chemical Regent Co., Shanghai, China) to dissolve the formazan crystals. The absorbances of the individual wells were determined at 570 nm with a microplate reader (Bio-Rad, Sunnyvale, CA, USA).

### Apoptosis assay

Cells were seeded in six-well plates. Twelve hours later, the cells were treated with different concentrations of resveratrol or control medium for 48 h. Fluorescein Annexin V-fluorescein isothiocyanate (FITC) and propidium iodide (PI) double labeling was performed with an Annexin V-FITC/PI apoptosis detection kit (Beyotime, Haimen, China) in accordance with the manufacturer’s instructions. The percentage of apoptotic cells was determined by flow cytometry analysis on a BD FACSCalibur™ flow cytometry system (BD Biosciences, Franklin Lakes, NJ, USA).

### Plasmids

For gene knockdown experiments, *c-Myc* siRNA (sense: AUGAUGUUUU UGAUGAAGGUC; anti-sense: CCUUCAUCAAAAACAUCAUCA) and negative control siRNA were obtained from GenePharma (Shanghai, China). For gene overexpression vector construction, the open reading frames and downstream 3′-UTR of *c-Myc* were cloned into the pcDNA3.1+ vector (Invitrogen) between the HindIII and EcoRI sites and driven by the cytomegalovirus promoter. For the luciferase reporter assay, the 3′-UTR fragment of *MICA* or *MICB* was amplified and cloned into the XhoI and NotI sites downstream of the SV40 promoter-driven Renilla luciferase cassette in the psiCHECK-2 plasmid (Promega, Madison, WI, USA). A Fast Mutagenesis kit (Vazyme Biotech, Nanjing, China) was used to mutate the miR-17 binding sites in the *MICA* or *MICB* 3′-UTR vectors according to the manufacturer’s instructions.

### Transfection and drug treatments

BCap37 and MDA-MB-231 cells were seeded in 6-cm cell culture dishes at 1.6×10^5^ and 1.2×10^5^ cells/mL, respectively. The cells were transfected with a 50 nM miR-17 mimic, miR-17 inhibitor or the corresponding scrambled NC (Ribobio, Guangzhou, China) with lipofectamine 2000 (Invitrogen) per the manufacturer’s instructions. Twenty-four hours later, the medium was replaced and the cells were prepared for subsequent experiments.

Resveratrol was purchased from Selleck Chemicals (Houston, TX, USA) and stored at −20°C. It was dissolved in DMSO to a 109.5 mM stock. Cells were treated with 6.25, 25, 50 or 100 μM resveratrol for 48 h.

### RNA extraction and quantitative real-time PCR (qRT-PCR) analysis of mRNA and miRNA

Total RNA was isolated from breast cancer cells with RNAiso Plus (TaKaRa, Kusatsu, Japan). The extracted RNA was synthesized into cDNA with a PrimeScriptTM RT reagent Kit (#RR037A, TaKaRa) to produce a template suitable for qRT-PCR. For mRNA reverse-transcriptase PCR (RT-PCR), random hexamer primers were used, and for miRNA RT-PCR, specific primers (Ribobio) were used.

qRT-PCR was used to detect the expression of *MICA*, *MICB*, *pri-miR-17-92* and *c-Myc*, with hypoxanthine phosphoribosyltransferase 1 (*HPRT1*) as an internal normalized reference. The sequences of the PCR primers are listed in Supplementary Table 4 Mature miRNA expression was quantified by qRT-PCR with specific BulgeLoop miRNA qRT-PCR primers (Ribobio), with *U6* small nuclear RNA as an internal normalized reference (Supplementary Table 5). qRT-PCR was performed in a LightCycler 480II system (Roche Diagnostics, Basel, Switzerland) with a SYBR Premix EX Tag kit (#RR420A, TaKaRa). Relative expression was calculated as 2^-ΔCt^ or 2^-ΔΔCt^ after normalization with the reference control.

### Flow cytometry analysis

For cell-surface protein expression analysis, cells were harvested, washed with PBS, and incubated with antibodies for 25 min at 4°C in the dark. Data acquisition and analysis were carried out on a BD FACSCalibur™ flow cytometry system with the CellQuest software package (BD Biosciences). The following antibodies were purchased from R&D systems (R&D, Minneapolis, MN, USA): MICA-PE (FAB1300P-100), MICB-PE (FAB1599P-025), and mouse IgG2B PE-conjugated antibody (IC0041P). The increase in mean fluorescence intensity (ΔMFI) was calculated as: (MFI with specific mAb – MFI with isotype control) / MFI with isotype control. The relative MFI (rMFI) was employed to illustrate the difference between the ΔMFI of a specific treatment and that of the control, and was calculated as: ΔMFI of specific treatment / ΔMFI of control treatment [50].

### Bioinformatic analysis

MiRNAs that bind to the 3′-UTRs of *MICA* and *MICB* were predicted with different bioinformatic algorithms from various databases, including TargetScan (http://www.targetscan.org/), miRanda (http://www.microrna.org/microrna/home.do), RNA22 (https://cm.jefferson.edu/rna22/), miRWalk (http://www.umm.uni-heidelberg.de/apps/zmf/mirwalk/), and Starbase (http://starbase.sysu.edu.cn/targetSite.php). If a miRNA was predicted to bind to the 3′-UTR of a certain gene, it received a score of “1”; otherwise, it received a score of “0”. The miRNAs with the highest aggregate scores were chosen for further experiments.

TCGA (https://cancergenome.nih.gov/) provides breast cancer patient mRNA and miRNA sequence results. All the available level-3 data (1099 breast cancer samples) with detailed miR-17 and *MICA/B* information were extracted from the data portal of TCGA and used as input for correlation analysis by the R package edgeR [51].

### Luciferase reporter assay

BCap37 cells were cultured in 96-well plates at a density of 2×10^4^ cells per well. The cells were co-transfected with a 50 nM miR-17 mimic or NC, and 50 ng of the *MICA* or *MICB* 3′-UTR wild-type or mutant psiCHECK-2 reporter plasmid (Promega). The transfection was performed with Lipofectamine 2000 according to the manufacturer’s instructions. After 48 h of transfection, a luciferase assay was performed with a Dual Luciferase Reporter Assay System Kit (Promega) as described in the manufacturer’s protocol. Luminometry readings were obtained with a Varioskan Flash Spectral Scanning Multimode Reader (Thermo Scientific, Waltham, MA, USA). Firefly luciferase activity was normalized to constitutive Renilla luciferase activity. Each transfectant was assayed in triplicate.

### Cytotoxicity assay *in vitro*

After pretreatment with 6.25 μM or 25 μM resveratrol for 48 h, BCap37 cells were used as target cells and seeded into a round-bottom 96-well plate at a density of 1×10^4^ cells per well. Cells from the NK cell line NK-92MI were pretreated with PBS or anti-NKG2D antibodies (50 mg/mL, Novus Biologicals, Littleton, CO, USA) for 1 h before the cytotoxicity assay, and then were added to the wells at effector-to-target ratios of 10:1, 5:1 and 2.5:1. After a 4-h co-incubation at 37°C in a humidified atmosphere of 5% CO_2_, the supernatants were harvested and analyzed by the CytoTox 96 Non-Radioactive Cytotoxicity Assay (Promega). The cytotoxicity of the effector cells against the target cells was assessed with the following equation: Cytotoxicity = (Experimental - Effector spontaneous - Target spontaneous) / (Target maximum - Target spontaneous) ×100%.

### Lung clearance assay

Male C57BL/6 mice (8-9 weeks old) were divided into three groups and intraperitoneally injected with PBS, anti-mouse NKG2D monoclonal antibodies (300 μg per mouse, #191004, Novus Biologicals) or anti-NK1.1 antibodies (300 μg per mouse, #108712, Biolegend, San Diego, CA, USA), respectively. Twenty-four hours later, HeLa cells (which are not efficiently killed by mouse NK cells, and thus served as an internal control) were labeled with PKH26 (MINI26-1KT, Invitrogen), and various BCap37 cells (pretreated with 6.25 μM, 25 μM resveratrol or control for 48 h) were labeled with CFSE (C34554, Invitrogen). Stained cells (5×10^6^ cells of each population) were mixed in 1 mL PBS, and 0.4 mL of this mixture was injected into the tail vein of each mouse. Five hours later, the lungs were harvested and single-cell suspensions were prepared for flow cytometry. The ratio of tested target cells to HeLa cells was calculated. In each group, the ratio of control-medium-pretreated BCap37 cells to HeLa cells was set as 100% [13, 52].

### Immunohistochemistry in a xenograft model

BCap37 cells (2×10^6^ cells in 0.2 mL PBS) were subcutaneously implanted into the right hind flanks of female BALB/c (nu/nu) mice. The length and width of the tumor were measured every three days with a caliper, and the tumor volume was calculated as (length × width × width × 0.5). When the tumor size was approximately 40 mm^3^, the mice were divided into three groups. Two groups were given resveratrol (dissolved in DMSO: polyethylene glycol 400: distilled and deionized water [1:1:3]) intraperitoneally at a daily dose of 25 or 100 mg/kg for four consecutive weeks, whereas the control group received the vehicle only [53]. Body weights and tumor volumes were recorded every week. After four weeks of treatment, the mice were sacrificed. The tumors were fixed in 4% paraformaldehyde (Sinopharm Chemical Reagent Co., Shanghai, China) and sectioned. The slides were stained with hematoxylin and eosin (Hematoxylin and Eosin Staining Kit, C0105, Beyotime Biotechnology, Shanghai, China) so that cell types and morphologic changes could be observed. Specific antibodies against MICA/B (#Ab54413, Abcam, Cambridge, MA, USA) were used for immunohistochemical staining. The slides were then incubated with a goat anti-rabbit IgG/horseradish peroxidase (HRP) complex (PV-6001, ZSGB-BIO, Beijing, China) for 45 minutes at room temperature. After being washed twice in PBS, the sections were incubated with HRP-conjugated streptavidin (DAB kit, ZLI-9019, ZSGB-BIO) for 30 minutes and then washed twice in PBS. Positive results were visualized with 3, 3′-diaminobenzidine. The results were analyzed by observation under a microscope, and images were acquired at ×400 magnification.

### Western blot

After treatment with resveratrol or transfection with siRNA or expression vectors, BCap37 cells were harvested. Total proteins were extracted in RIPA lysis buffer (Beyotime Institute of Biotechnology, China) plus 1 mM phenylmethylsulfonyl fluoride and a 1x protease inhibitor cocktail (Sigma-Aldrich, Sigma, MO, USA). The protein content was detected with a bicinchoninic acid (BCA) kit (Thermo Fisher Scientific, MA, USA). Then, 40 ug lysate was run on a 10% polyacrylamide gel and transferred onto polyvinylidene fluoride membranes (Roche, Switzerland). The membranes were probed with specific antibodies against c-Myc (32072, Abcam) and β-Actin (A2228, Sigma-Aldrich). β-Actin was adopted as an internal control. Signals from HRP-conjugated secondary antibodies were detected with enhanced chemiluminescence reagents (Thermo Scientific, Darmstadt, Germany) according to the manufacturer’s instructions. Blots were scanned with a ChemiDoc Touch Imaging System (Bio-Rad, CA, USA). Experiments were repeated independently at least three times.

### Statistical analysis

All data are presented as mean ± standard error of the mean (SEM) from at least three independent experiments. Statistical significance was assessed with Student’s t-test or one-way analysis of variance. Overall survival was defined as the time period between diagnosis and disease-related death. Overall survival was censured when the patient either was alive at the last follow-up or died from causes unrelated to the disease. Overall survival probability rates were calculated by the Kaplan–Meier method, and compared through log-rank test univariate analysis. Linear correlation and Pearson’s correlation were used to evaluate the correlation between two variants. Statistical analyses were performed with GraphPad Prism Ver. 5.01 (San Diego, CA, USA) in accordance with the guidelines for the software. Statistical significance was set at *p* < 0.05 and displayed as *** for *p*<0.001; ** for *p*<0.01 and * for *p*<0.05.

## SUPPLEMENTARY MATERIALS FIGURES AND TABLES



## Abbreviations

MICA: Major histocompatibility complex class I chain-related protein A
MICB: Major histocompatibility complex class I chain-related protein B
NKG2D: Natural killer group 2 member D
NKG2DL: Natural killer group 2 member D ligand
3′-UTR: 3′-untranslated region
NC: negative control
OS: overall survival
TCGA: The Cancer Genome Atlas
