# Supplementary material for: Resveratrol promotes MICA/B expression and natural killer cell lysis of breast cancer cells by suppressing c-Myc/miR-17 pathway
Source: Oncotarget. 2017 Jul 22;8(39):65743–58. doi: 10.18632/oncotarget.19445 (PMC5630368; doi:10.18632/oncotarget.19445)
Supplement: Supplementary file 1 [file oncotarget-08-65743-s001.pdf]

Resveratrol promotes MICA/B expression and natural killer cell lysis of breast cancer cells by suppressing c-Myc/miR-17 pathway

SUPPLEMENTARY MATERIALS

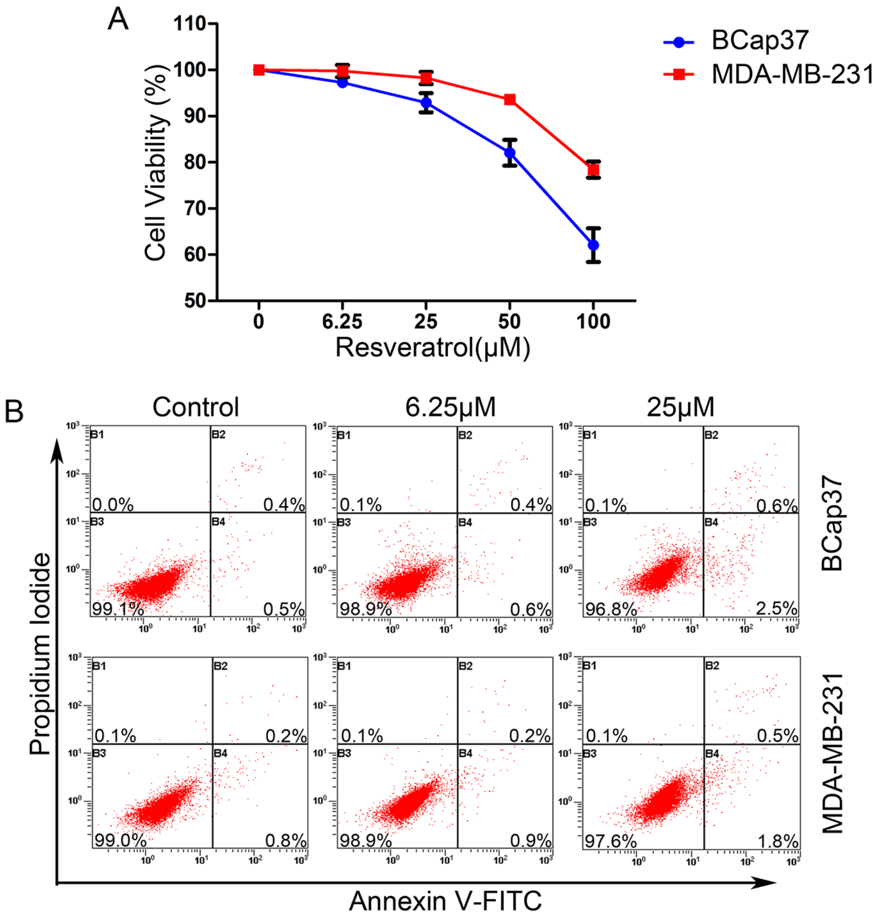

Supplementary Figure 1: Effects of resveratrol on cell viability and apoptosis. Breast cancer cells were treated with various concentrations of resveratrol or control medium for 48 h. **(A)** The effect of resveratrol on cell viability was determined with an MTT assay. **(B)** Flow cytometric analysis of the PI- and Annexin V-FITC-stained apoptotic population after 48-h exposure to resveratrol. Error bars represent the SEM obtained from three independent experiments.

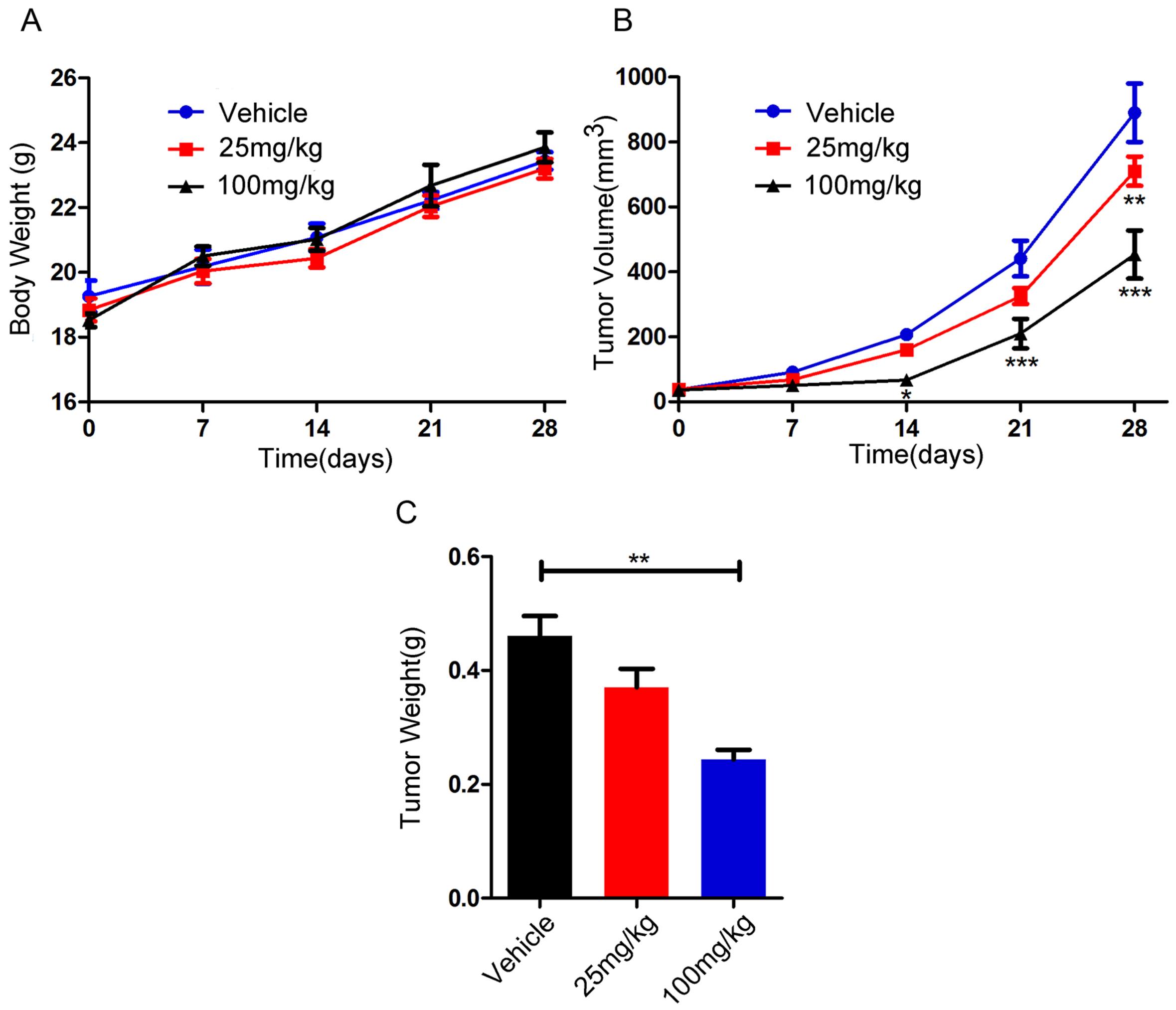

Supplementary Figure 2: BCap37-cell tumor growth is inhibited by resveratrol. (A, B) BCap37 cells were subcutaneously implanted into the right hind flanks of female BALB/c (nu/nu) mice. During the 28-day administration of resveratrol or vehicle, the body weights of the mice (A) and tumor volumes (B) were measured every week. **(C)** After 28-day administration of resveratrol or vehicle, mice were sacrificed, and tumor weights were measured. Error bars in this figure represent the mean ± SEM. *P<0.05, **P<0.01, ***P<0.001.

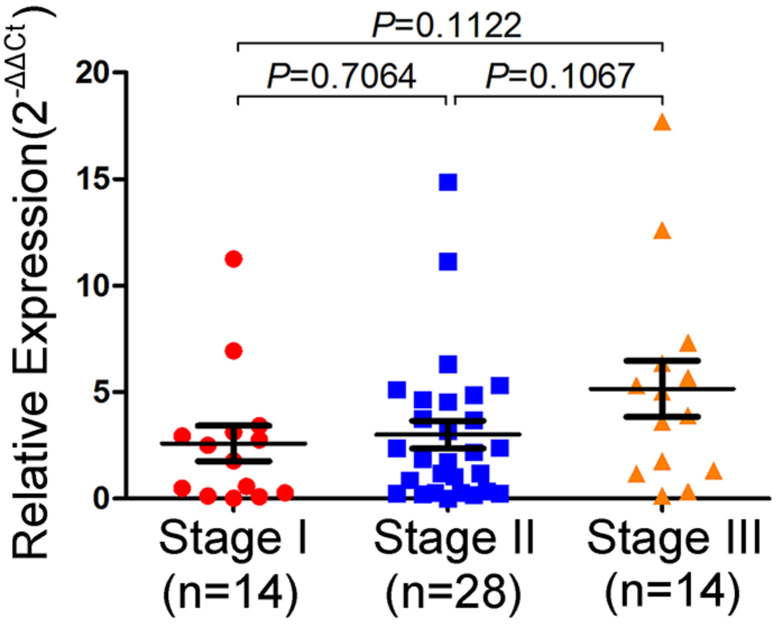

Supplementary Figure 3: The correlation between miR-17 expression and tumor stages. The levels of miR-17 were compared among TNM stage-I, -II, and -III groups. Error bars in this figure represent the mean ± SEM.

Supplementary Table 1: The results of bioinformatic prediction and scoring of miRNA binding to the *MICA* 3′-UTR

Gene	MicroRNA	TargetScan	Miranda	RNA22	miRWalk	Starbase	SUM
MICA	MiR-17	1	1	1	0	1	4
	MiR-18a	0	0	1	0	0	1
	MiR-19a	1	0	0	0	0	1
	MiR-20a	1	1	1	0	1	4
	MiR-19b	1	0	0	0	0	1
	MiR-92a	1	0	0	0	0	1

Supplementary Table 2: The results of bioinformatic prediction and scoring of miRNA binding to the *MICB* 3′-UTR

Gene	MicroRNA	TargetScan	Miranda	RNA22	miRWalk	Starbase	SUM
MICB	MiR-17	1	1	0	1	1	4
	MiR-18a	0	0	0	0	0	0
	MiR-19a	0	0	0	0	0	0
	MiR-20a	1	1	0	1	1	4
	MiR-19b	0	0	0	0	0	0
	MiR-92a	0	0	0	0	1	1

Supplementary Table 3: Patients' baseline characteristics, and the relationship between miR-17 levels and clinical features

Baseline characteristics	Total (N=56)	MiR-17 levels[a] High (N=28)	MiR-17 levels[a] Low (N=28)	P-value[b]
Mean age (SD), y	53.8 (13.1)	54.7 (12.7)	51.3 (13.9)	
Histological type, N (%)				
Ductal	29 (51.8)	15 (51.7)	14 (48.3)	
Lobular	6 (10.7)	3 (50.0)	3 (50.0)	
Other	21 (37.5)	10 (47.6)	11 (52.3)	
TNM stage, N (%)				
I	14 (25.0)	7 (50.0)	7 (50.0)	
II	28 (50.0)	12 (42.9)	16 (57.1)	
III	14 (25.0)	9 (64.3)	5 (35.7)	
ER expression, N (%)				
Negative	31 (55.4)	14 (45.2)	15 (54.8)	
Positive	25 (44.6)	14 (56.0)	13 (44.0)	
PR expression, N (%)				
Negative	27 (48.2)	15 (55.6)	12 (44.4)	
Positive	29 (51.8)	13 (44.8)	16 (55.1)	
HER2 expression, N (%)				
Negative	36 (64.3)	17 (47.2)	19 (52.8)	
Positive	20 (35.7)	11 (55.0)	9 (45.0)	
Lymph node metastases, N (%)				
Yes	26 (46.4)	15 (57.7)	11 (42.3)	
No	30 (53.6)	13 (43.3)	17 (56.7)	
Outcomes				
Mean follow-up duration (SD), y	42.4 (17.9)	39.9 (17.7)	45 (17.6)	
Cancer relapse, N (%)				0.015*
Yes	26 (46.4)	18 (69.2)	8 (30.8)	
No	30 (53.6)	10 (33.3)	20 (66.7)	
Death, N (%)				0.026*
Yes	21 (37.5)	15 (71.4)	6 (28.6)	
No	35 (62.5)	13 (37.1)	22 (62.9)	

Nonsignificant P values have not been reported. * Significant difference ($p<0.05$).

[a]The median value in all cases was adopted as the threshold value

[b]Fisher's exact test for categorical variables; t test for continuous variables.

ER, estrogen receptor; PR, progesterone receptor; HER2, human epidermal growth factor receptor 2

Supplementary Table 4: Primers used for mRNA qRT-PCR

Transcript	Sequences (from 5'-3')
MICA	F: ACAATGCCCCAGTCCTCCAGA
	R: ATTTTAGATATCGCCGTAGTTCCT
MICB	F: TGAGCCCCACAGTCTTCGTTAC
	R: TGCCCTGCGTTTCTGCCTGTCATA
Pri-miR-17-92	F: CAGTAAAGGTAAGGAGAGCTCAATCTG
	R: CATACAACCACTAAGCTAAAGAATAATCTGA
c-Myc	F: CCCTCCACTCGGAAGGACTA
	R: GCTGGTGCATTTTCGGTTGT
HPRT1	F: TGACACTGGCAAAACAATGCA
	R: GGTCCTTTTCACCAGCAAGCT

Supplementary Table 5: Primers used for miRNA qRT-PCR

Transcript	miRBase Accession
hsa-miR-17	MI0000071
hsa-miR-20a	MI0000076
U6	F: CTCGCTTCGGCAGCACATA
	R: AACGCTTCACGAATTTGCG
